# Enzyme‐activatable dual‐locked fluorescent probe for precision imaging of cutaneous squamous cell carcinoma

**DOI:** 10.1002/smo2.70018

**Published:** 2025-09-25

**Authors:** Yanhua Li, Shan Zuo, Yushi Chen, Junliang Zhou, Ling Shi, Lin Yuan

**Affiliations:** ^1^ State Key Laboratory of Chemo and Biosensing College of Chemistry and Chemical Engineering Hunan University Changsha Hunan China; ^2^ MOE Key Laboratory for Analytical Science of Food Safety and Biology Fujian Provincial Key Laboratory of Analysis and Detection Technology for Food Safety College of Chemistry Fuzhou University Fuzhou Fujian China

**Keywords:** cutaneous squamous cell carcinoma, disease diagnosis, dual‐locked probes, enzyme, fluorescent probes

## Abstract

Cutaneous squamous cell carcinoma (cSCC), the second most common skin cancer, requires early and accurate detection to optimize clinical management. Fluorescence imaging has emerged as a non‐invasive and cost‐effective tool with high sensitivity for tumor diagnosis; however, existing probes for cSCC are limited in achieving rapid and specific imaging. In this study, we introduce **FC‐1**, a dual‐tandem activatable fluorescent probe designed for precise cSCC diagnosis by simultaneously targeting fibroblast activation protein α and cathepsin C (CTSC). This dual‐locked activation strategy effectively reduces nonspecific signals in normal tissues while enhancing diagnostic specificity. In vivo studies in SCC‐7 xenografted mice demonstrated **FC‐1**'s ability to visualize subcutaneous tumors with high sensitivity and specificity. Moreover, **FC‐1** enabled clear differentiation between cSCC and keloids. The probe's dual‐activation mechanism and robust performance underscore its potential as a clinically translatable tool for early cSCC detection and differential diagnosis.

## Graphical abstract

FC‐1 is the first dual‐locked fluorescent probe developed for imaging cutaneous squamous cell carcinoma (cSCC), utilizing a dual‐enzyme activation mechanism to markedly improve imaging specificity. By simultaneously targeting FAPα and CTSC, FC‐1 effectively minimizes background fluorescence in normal tissues. In SCC‐7 xenograft mouse models, FC‐1 enabled highly sensitive and specific visualization of subcutaneous cSCC tumors.

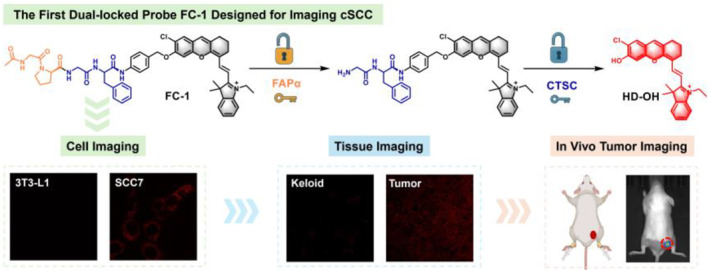

## 1 INTRODUCTION

Cutaneous squamous cell carcinoma (cSCC) is the second most prevalent form of skin cancer. Some studies have suggested that the global incidence of cSCC cases was 2.4 million in 2019.^[1]^ Recent studies have indicated a significant increase in the prevalence of cSCC in the United States over the past 3 decades, with prevalence rising from 50% to 200%.[^2^, ^3^, ^4^] cSCC is a malignancy that results from the transformation of keratin‐forming cells of the epidermis into malignant cells. Clinically, it typically manifests as a sclerotic crusted lesion.^[5]^ cSCC is prone to occur on any skin surface exposed to ultraviolet radiation or pre‐existing chronic keloids, chronic ulcers, or burn wounds.^[6]^ While the majority of cSCC can be successfully treated through surgical resection, a subset of cSCC is characterized by high recurrence and metastasis. It has been reported that a subset of cSCC patients experience local recurrence and metastasis in 3%–5% of cases following complete resection of the primary tumor.[^3^, ^7^, ^8^] Therefore, the prompt identification of aggressive cSCCs can guide subsequent investigations and treatments.

Currently, the diagnosis of cSCC relies on the physician's interpretation of clinical information and is subsequently confirmed through invasive skin biopsy.^[9]^ Although histopathology remains the gold standard for diagnosing cSCC, several non‐invasive optical techniques are now utilized in the diagnostic process. These techniques include dermoscopy, reflectance confocal microscopy (RCM), and high‐frequency ultrasonography (HFUS).^[10]^ However, these techniques exhibit several notable drawbacks, including limited imaging depth, reduced spatio‐temporal resolution, susceptibility to operator‐dependent variability, and constrained imaging precision, thereby potentially compromising their diagnostic accuracy. Advancements in fluorescence imaging technology have established it as a promising modality owing to its non‐invasive nature, real‐time monitoring capabilities, cost‐effectiveness, exceptional sensitivity, and high spatial‐temporal resolution, leading to its increasing application in tumor diagnosis.[^11^, ^12^, ^13^, ^14^] Although activatable probes have been reported for tumor detection,[^15^, ^16^] demonstrating good sensitivity and specificity, activatable probes capable of achieving rapid imaging of cSCC are rarely reported.[^17^, ^18^, ^19^] Therefore, there exists a need to develop novel fluorescent probes to achieve early and accurate detection of cSCC.

Fibroblast activation protein α (FAPα), a membrane‐anchored type II transmembrane serine protease,^[20]^ is highly overexpressed in more than 90% of epithelial tumors, whereas its expression is significantly lower in normal human tissues.^[21]^ FAPα has been implicated in tumor growth, metastasis, the promotion of extracellular matrix degradation, and cellular invasiveness during tumor progression.^[22]^ Therefore, it could be regarded as a promising biomarker for cSCC diagnosis. However, FAPα exhibits significant overexpression in keloids,^[23]^ which commonly arise following cutaneous injuries, including burns, surgical procedures, and piercings.^[24]^ Given that keloids may generate “false‐positive signals” in physiological contexts, the use of single‐locked probes designed for FAPα recognition poses a heightened risk of misdiagnosis during cSCC diagnosis. Cysteine cathepsin proteases play a critical role in tumorigenesis, particularly in SCC, with studies demonstrating that their overexpression is strongly associated with disease aggressiveness and metastatic potential.[^25^, ^26^] Notably, studies have observed that both the expression levels and enzymatic activity of cathepsin C (CTSC) exhibit significant heterogeneity in SCC tissues compared to normal tissues.^[27]^ Given the intrinsic differences in CTSC levels between normal tissues and SCC, fluorescence imaging via dual‐target activation of CTSC and FAPα provides a rationale for designing a “dual‐locked” activatable fluorescent probe to enhance diagnostic specificity for cSCC, thereby enabling more precise discrimination between cSCC and surrounding non‐malignant tissues.

Herein, we present a dual‐enzyme activatable fluorescent probe, termed **FC‐1**, for the differential diagnosis of cSCC. **FC‐1** incorporates a dual‐activation mechanism targeting both FAPα and CTSC recognition units, designed to enhance diagnostic specificity for cSCC. In vivo bioimaging studies of **FC‐1** in mice demonstrated that probes based on the dual‐locked strategy effectively avoided nonspecific activation in normal tissues. Benefiting from the dual‐activation strategy, **FC‐1** demonstrated significantly higher sensitivity and specificity, enabling precise differentiation of cSCC from keloids. Furthermore, **FC‐1** demonstrated successful application in visualizing subcutaneous tumors in SCC7 xenografted mice. We believe that this novel diagnostic approach holds promising clinical implications for the specific diagnosis of cSCC.

## 2 RESULTS AND DISCUSSION

### 2.1 Rational design of the probes

**SCHEME 1:**
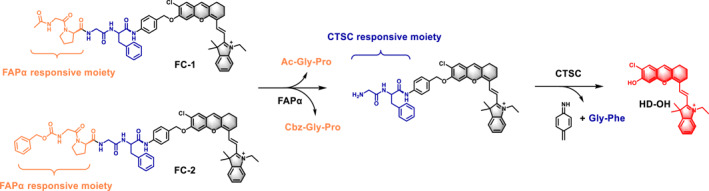
Illustration of the sensing mechanism of probes **FC‐1** and **FC‐2** in response to fibroblast activation protein α (FAPα) and CTSC.

Compared with single‐locked probes, enzyme‐activated dual‐locked probes significantly reduce the incidence of false‐positive signals, address the issue of low specificity inherent to biologically active substances, and enable precise imaging.[^28^, ^29^, ^30^, ^31^] Consequently, we developed a dual‐enzyme near‐infrared (NIR) fluorescent probe capable of being specifically and sequentially activated by FAPα and CTSC. As illustrated in Scheme 1 and Supporting Information S1: Scheme S1, the developed probes (**FC‐1** and **FC‐2**) comprise two principal components: a tetrapeptide (Ac‐Gly‐Pro‐Gly‐Phe or Cbz‐Gly‐Pro‐Gly‐Phe) serving as the recognition unit, which selectively targets FAPα and CTSC, and a NIR hemicyanine dye (HD‐OH) functioning as the fluorescent reporter.[^32^, ^33^] These components are linked by a self‐immolative 4‐aminobenzyl alcohol (PABA) spacer. In the intact probe, the intrinsic intramolecular charge transfer (ICT) process of HD‐OH is suppressed, resulting in quenched fluorescence.[^32^, ^33^] Following specific enzymatic cleavage by FAPα, the probe undergoes initial hydrolysis to generate Gly‐Phe‐PABA‐HD, a non‐fluorescent intermediate. Subsequent proteolytic activation by CTSC removes the dipeptide Gly‐Phe, initiating a 1,6‐self‐elimination cascade of the PABA spacer.^[34]^ This structural rearrangement exposes the hydroxyl group of the hemicyanine fluorophore (HD‐OH), which undergoes deprotonation under physiological conditions to form an electron‐donating phenolate anion. This deprotonation enhances the ICT process, thereby restoring NIR fluorescence emission. Moreover, we constructed a single‐locked probe (**F‐1**) as a reference by conjugating a hemicyanine dye (HD‐OH) with the FAPα recognition moiety (Cbz‐Gly‐Pro) (Supporting Information S1: Scheme S2).

### 2.2 Spectral properties of the probes

**FIGURE 1:**
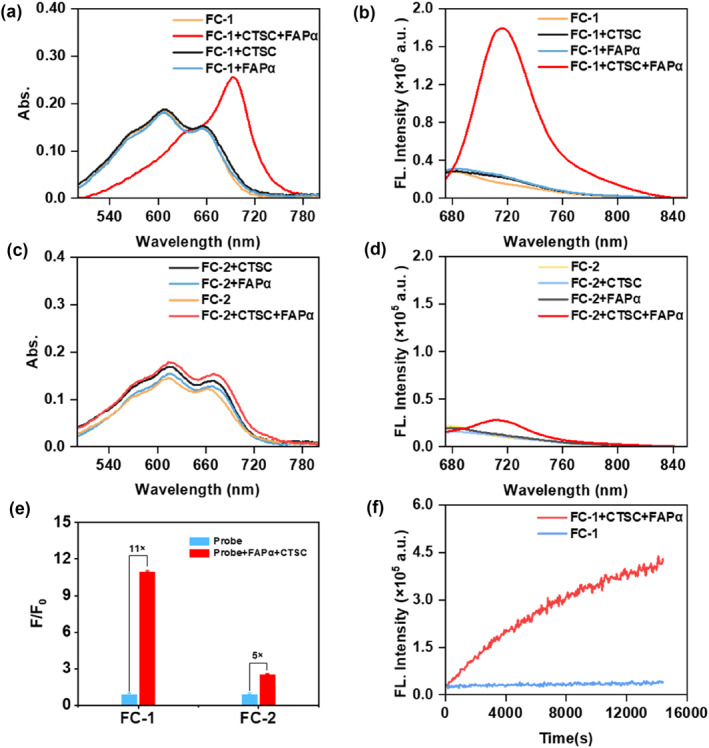
UV/Vis absorption (a, c) and fluorescence (b, d) spectra of 5 μM **FC‐1** or **FC‐2** were recorded in the absence and presence of fibroblast activation protein α (FAPα) (1.2 mU/mL) and CTSC (500 mU/mL) after incubation for 3 h at 37°C in HEPES buffer (50 mM, pH 7.4), respectively. (e) Quantitative comparison of fluorescence intensity enhancement ratios for **FC‐1** and **FC‐2** following enzymatic activation by FAPα and CTSC. (f) Time‐dependent fluorescence intensity of **FC‐1** (5 μM) was monitored over 4 h at 37°C in HEPES buffer (50 mM, pH 7.4) in the absence and presence of FAPα and CTSC.

To validate the dual‐enzyme‐activatable performance of **FC‐1** and **FC‐2**, we systematically evaluated their NIR absorption and fluorescence emission profiles in response to sequential activation by FAPα and CTSC. As illustrated in Figure 1a,b, **FC‐1** exhibited two absorption peaks at 600 and 645 nm, alongside a weak fluorescence emission at 680 nm. In the presence of FAPα and CTSC, the absorption peak of **FC‐1** at 600 nm was diminished, and a new absorption peak emerged at 690 nm. Moreover, after incubation with FAPα and CTSC, the fluorescence wavelength of **FC‐1** is red‐shifted to 715 nm, and its intensity is enhanced by 11‐fold (Figure 1e), indicating a robust fluorescence response to both enzymes. However, when FAPα or CTSC was added individually, the fluorescence intensity of **FC‐1** did not exhibit significant changes. In contrast, **FC‐2** displayed only a slight change in its absorption and fluorescence wavelength and intensity following incubation with FAPα and CTSC for 3 h (Figure 1c,d). These results revealed that, compared with **FC‐2**, **FC‐1** demonstrated a significantly stronger responsiveness to FAPα and CTSC. This disparity was likely due to the distinct affinities of **FC‐1** and **FC‐2** for FAPα. Additionally, we investigated the fluorescence response of the reference probe **F‐1** to FAPα. After incubation with FAPα, the fluorescence intensity of **F‐1** at 715 nm was enhanced by 19‐fold (Supporting Information S1: Figure S1).

Subsequently, the stability of **FC‐1** at physiological temperature (37°C) and pH (7.4) was assessed. The fluorescence of **FC‐1** at 715 nm showed a negligible increase (<5%) over a four‐hour period, indicating the probe's superior stability (Figure 1f). In contrast, following incubation with FAPα and CTSC, **FC‐1** demonstrated a time‐dependent increase in fluorescence intensity (approximately 11.7‐fold over 4 h), validating the enzyme‐specific activation of the probe.

**FIGURE 2:**
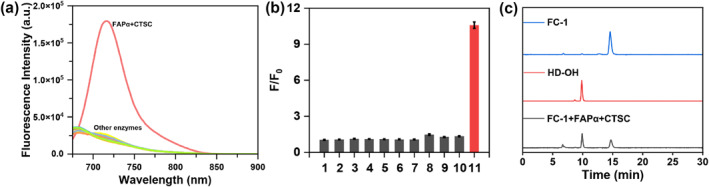
Fluorescence spectra (a) and intensity (b) after 3 h incubation with other analytes in a HEPES buffer (50 mm, pH 7.4). (1) **FC‐1** (5 μM); (2) alkaline phosphatase (ALP) (50 U/L); (3) NOQ1 (500 ng/mL); (4) Caspase‐3 (75 ng/mL); (5) CTSB (75 U/L); (6) ARS (9 U/L); (7) GrzB (0.5 U/mL); (8) LTA4H (1.0 μg/mL); (9) CTSC (500 mU/mL); (10) FAPα (1.2 mU/mL); (11) FAPα (1.2 mU/mL) + CTSC (500 mU/mL). Ex = 660 nm, Em = 715 nm. (c) HPLC analysis of **FC‐1** (blue), HD‐OH (red), **FC‐1** + FAPα + CTSC (black).

We next investigated the selectivity of the probes for various enzymes and other analytes, such as quinone dehydrogenase 1 (NQO1), cathepsin B (CTSB), caspase‐3, alkaline phosphatase (ALP), arylsulphatase (ARS), granzyme B (GrzB), leukotriene A4 hydrolase (LTA4H). As depicted in Figure 2a,b, probe **FC‐1** demonstrated minimal fluorescence enhancement in the presence of biomolecules other than FAPα and CTSC. These findings confirm that **FC‐1** exhibits exceptional selectivity for the simultaneous detection of FAPα and CTSC, underscoring its potential as a specific diagnostic tool for these enzymatic targets.

Furthermore, we confirmed the activation mechanism of **FC‐1** by FAPα and CTSC via HPLC analysis. As shown in Figure 2c, the retention time of **FC‐1** was 14 min, whereas the retention time of HD‐OH was 10 min. When the mixed solution of **FC‐1** with FAPα and CTSC was incubated at 37°C for 2 hours, the decomposition product HD‐OH was observed, confirming enzymatic cleavage of the probe by FAPα and CTSC and subsequent exposure of the fluorophore's hydroxyl group.

### 2.3 Fluorescence imaging of endogenous FAPα and CTSC in living cells

As validated in vitro, these collective findings confirm that **FC‐1** undergoes sequential enzymatic activation by FAPα and CTSC, leading to fluorescence activation. The validated mechanism prompted further investigation through cellular level studies to assess its bioactive potential within in vitro and in vivo models. To evaluate the ability of **FC‐1** to enable live‐cell imaging, its cytotoxicity was first assessed in SCC7 cells using the MTT assay, a widely used method for evaluating cell viability. As shown in Supporting Information S1: Figure S2, cell viability remained above 80% following treatment with 20 μM **FC‐1**, demonstrating the probe's low cytotoxicity and excellent biocompatibility.

**FIGURE 3:**
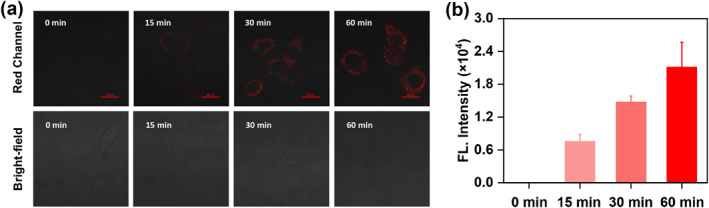
(a) Confocal fluorescence image of the SCC7 cells incubated with **FC‐1** (5 μM) for varying durations. *λ*_ex_ = 640 nm; *λ*_em_ = 663−738 nm. Scale bar: 20 μm. (b) Quantification of the relative fluorescence intensities from (a).

The SCC7 cell line, a murine‐derived SCC model, was selected to evaluate the enzymatic activation (FAPα and CTSC) potential of the probe in live cells. To assess the functional applicability of **FC‐1** in a cellular context, we conducted real‐time live‐cell imaging to simultaneously monitor intracellular FAPα and CTSC activity in SCC7 cells. As demonstrated in Figure 3, SCC7 cells incubated with **FC‐1** for varying durations (15, 30, and 60 min) exhibited red fluorescence emission in the red channel, with fluorescence intensity peaking at 60 min. These findings confirm that **FC‐1** effectively visualizes SCC7 cells, highlighting its potential for time‐dependent live‐cell imaging applications.

**FIGURE 4:**
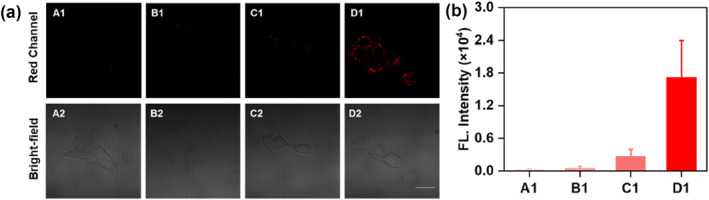
(a) Confocal fluorescence image of the SCC7 cells incubated with **FC‐1** (5 μM). (A) SCC7 cells were treated with SP13786 (10 μM) for 0.5 h, followed by co‐incubation with **FC‐1** (5 μM) for an additional 1 h. (B) SCC7 cells were pretreated with E‐64 (20 μM) for 1 h, followed by co‐incubation with **FC‐1** (5 μM) for an additional 1 h. (C) SCC7 cells were pretreated with E‐64 (20 μM) for 1 h and SP13786 (10 μM) for 0.5 h, followed by co‐incubation with **FC‐1** (5 μM) for an additional 1 h. (D) SCC7 cells were treated with **FC‐1** (5 μM) for 1 h. *λ*_ex_ = 640 nm; *λ*_em_ = 663−738 nm. Scale bar: 20 μm. (b) Quantification of the relative fluorescence intensities from (a).

To confirm that **FC‐1** activation is dependent on both FAPα and CTSC, four experimental groups were established: Group A (FAPα inhibition), in which SCC7 cells were pretreated with 10 μM SP13786 (a selective FAPα inhibitor^[35]^) for 0.5 h, and subsequently incubated with **FC‐1** for 1 h; Group B (CTSC inhibited) in which cells were pretreated with 20 μM E‐64 (a selective CTSC inhibitor^[36]^) for 1 h and subsequently incubated with **FC‐1** for another 1 h;. Group C (dual inhibition) cells were co‐treated with E‐64 (20 μM, 1 h) and SP13786 (20 μM, 0.5 h), then incubated with **FC‐1** for 1 h; and Group D (control), in which cells received **FC‐1** alone. Confocal imaging demonstrated robust fluorescence solely in Group D, whereas Groups A–C displayed markedly reduced fluorescence intensities (Figure 4). Collectively, these findings confirm that **FC‐1** requires sequential proteolytic activation by endogenous FAPα and CTSC, thereby allowing for spatiotemporal fluorescence imaging of their enzymatic activity in live cells.

**FIGURE 5:**
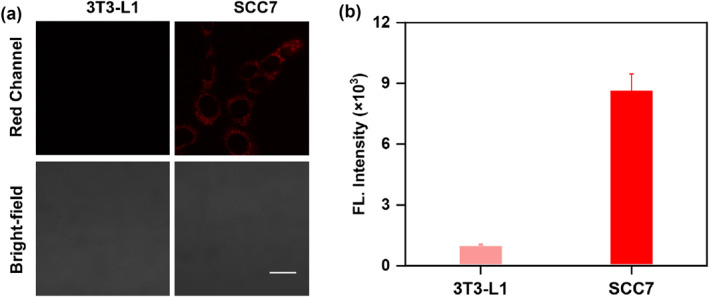
(a) Confocal fluorescence images of 3T3‐L1 or SCC7 cells incubated with **FC‐1** (5 μM). Scale bar: 20 μm. (b) Quantification of the relative fluorescence intensities from (a).

To evaluate the capacity of **FC‐1** to distinguish between SCC and normal cells, we evaluated its fluorescence imaging performance in SCC7 and 3T3‐L1 (murine fibroblast cell line) cells. As shown in Figure 5, SCC7 cells incubated with **FC‐1** exhibited a significant increase in NIR fluorescence intensity, whereas 3T3‐L1 cells displayed negligible fluorescence signal intensity under the same experimental conditions. These results collectively demonstrate that **FC‐1** specifically distinguishes between SCC7 and 3T3‐L1 cells based on their differential enzymatic activity.

### 2.4 In vivo tumor imaging

**FIGURE 6:**
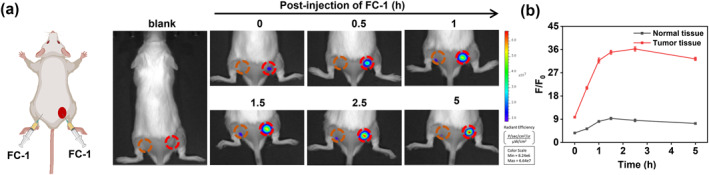
(a) Fluorescence images of tumor and normal tissues of SCC7 subcutaneous tumor‐bearing mice after intratumoral injection of probe **FC‐1** (100 μM, 50 μL). *λ*_ex_ = 710 nm; *λ*_em_ = 820–880 nm. (b) Quantification of the relative fluorescence intensities from (a).

Given **FC‐1**'s robust fluorescence imaging capabilities in vitro, we subsequently evaluated its in vivo tumor‐targeting diagnostic efficacy using cSCC tumor‐bearing mouse models. A subcutaneous cSCC tumor model was generated via injection of pre‐cultured SCC7 cells into the right hindlimb of BALB/c mice. Subcutaneous cSCC tumors were grown for 7 days prior to in vivo fluorescence imaging experiments. **FC‐1** was administered via subcutaneous injection into the left flank (designated as normal tissue) and intratumoral injection into the right flank (tumor‐bearing tissue). As shown in Figure 6, the fluorescence intensity at the tumor site increased rapidly post‐injection, peaking at a 3.6‐fold increase relative to baseline levels, whereas minimal signal variation was detected in normal tissue. These results confirm that **FC‐1** is selectively and enzymatically activated within tumor tissues, thereby facilitating the prospect of achieving high‐contrast in vivo imaging of cSCC in preclinical settings.

**FIGURE 7:**
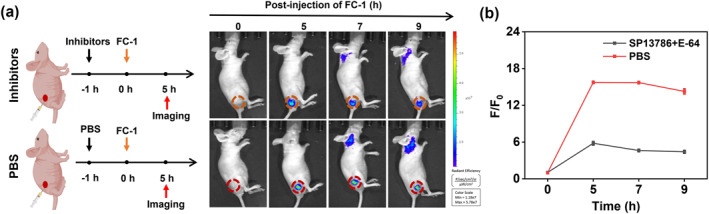
(a) SCC7 subcutaneous tumor‐bearing mice were pretreated with fibroblast activation protein α (FAPα) (SP13786, 10 μM, 50 μL) and CTSC (E‐64, 20 μM, 100 μL) inhibitors or PBS, followed by intratumoral injection of probe **FC‐1** (100 μM, 50 μL) for fluorescence imaging. *λ*_ex_ = 710 nm; *λ*_em_ = 820–880 nm. (b) Quantification of the relative fluorescence intensities from (a).

To determine whether **FC‐1** activation in tumor tissue is dependent on FAPα and CTSC, we performed pharmacological inhibition experiments in BALB/c nude mouse models of cSCC. Subcutaneous tumors were generated in mice through injection of SCC7 cells, and tumor‐bearing mice were grown over a 7‐day period prior to further analysis. In the experimental group, mice were pre‐treated with SP13786 (a FAPα inhibitor) and E‐64 (a CTSC inhibitor) via intravenous and intraperitoneal injections, respectively, followed by intratumoral administration of **FC‐1** after a 1‐h interval. The control group received PBS, followed by intratumoral administration of **FC‐1** under the same conditions. Longitudinal fluorescence imaging was performed over a 9‐h observation period to monitor probe activation dynamics. As shown in Figure 7a, the fluorescence intensity in the control group exhibited a rapid increase, peaking at a 16.4‐fold increase in signal intensity within tumor regions by 5 h post‐administration. In contrast, the inhibitor‐treated group exhibited only a 5.8‐fold increase. At the 5‐h time point, fluorescence signal intensity in the control group was 2.8‐fold higher than that in the inhibitor‐treated group, indicating a statistically significant difference (Figure 7b). These results collectively confirm that the tumor‐specific activation of **FC‐1** is mediated by the enzymatic activity of both FAPα and CTSC within the tumor microenvironment.

### 2.5 Discriminating cSCC from keloid tissue

**FIGURE 8:**
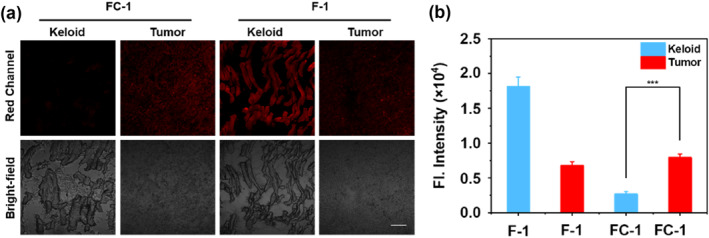
(a) Fluorescence imaging of keloid or tumor tissues incubated with probe **FC‐1** or **F‐1** (20 μM). Scale bar: 100 μm. (b) Quantification of the fluorescence intensities from (a). ***P ≤ 0.001.

Previous studies have demonstrated that FAPα is markedly overexpressed in keloid fibroblasts relative to normal fibroblasts, where it contributes critically to the pathological progression of wound border expansion observed in keloids.^[37]^ To validate the dual‐locked activation strategy of **FC‐1** in reducing nonspecific keloid tissue activation, a comparative fluorescence imaging analysis between **FC‐1** and the single‐locked **F‐1** was performed in cSCC and keloid tissue sections. The histological features of freshly excised cSCC and keloid tissues were characterized using hematoxylin and eosin (H&E) staining (Supporting Information S1: Figure S3). Freshly excised cSCC tissues and keloid tissue samples were cryosectioned into thin slices, treated with **FC‐1** or **F‐1** (20 μM for 1 h), and subsequently imaged via confocal laser scanning microscope (CLSM). As shown in Figure 8, both keloid and cSCC tissues incubated with **F‐1** exhibited strong fluorescence signals. In contrast, cSCC tissues displayed significantly higher fluorescence intensity than keloid tissues, highlighting **FC‐1**'s preferential activation in the tumor microenvironment. These findings confirm that **FC‐1** specifically targets tumor‐associated enzymatic activity (FAPα and CTSC) while minimizing nonspecific activation in non‐malignant keloid tissues, aligning with its dual‐locked design strategy.

## 3 CONCLUSION

In summary, **FC‐1**, a dual‐enzyme cascade‐activated probe, was designed to exploit the endogenous upregulation of FAPα and CTSC in cSCC, facilitating accurate differential diagnosis and therapeutic assessment of cSCC. **FC‐1** undergoes sequential enzymatic cleavage by FAPα and CTSC, yielding the fluorophore HD‐OH, and thereby demonstrating high selectivity for cSCC‐associated enzymatic activity. This probe facilitates real‐time in vivo imaging of cSCC tumors in mice and reliably distinguishes cSCC tissues from keloid tissues in ex vivo investigations. This “dual‐locked” design strategy guarantees the specific activation of **FC‐1** in the presence of both FAPα and CTSC, thereby enabling precise differentiation between cSCC tissues and keloid tissues. However, **FC‐1** also exhibits certain limitations, such as a relatively long response time. This delay is likely due to the sequential enzymatic cleavage required for activation in the tandem‐lock design. To overcome this, future designs may employ a parallel‐lock strategy, allowing independent and simultaneous activation by either enzyme, thereby achieving a faster response. Additionally, the in vivo signal‐to‐noise ratio remains suboptimal, indicating the need for further optimization of the probe design and performance. Overall, this approach holds considerable clinical potential for the early and specific detection of cSCC, thereby addressing a critical need in dermatologic oncology. For example, **FC‐1** could be formulated for topical administration, such as in the form of a gel or microneedle patch. These delivery strategies offer non‐invasive, patient‐friendly alternatives that may facilitate effective probe penetration and activation within superficial skin lesions. Such formulations would further enhance the clinical applicability of **FC‐1**, supporting its use in non‐invasive diagnostics or intraoperative visualization of cSCC.

## 4 EXPERIMENTAL METHODS

### 4.1 Synthesis of the probes

Details of the synthesis routes are shown in the Supporting Information S1: Schemes S1 and S2. The molecular structures of probes **FC‐1** and **FC‐2** were comprehensively characterized using NMR spectroscopy and MALDI‐TOF mass spectrometry (Figures S4‐S13).

### 4.2 Measurement of photophysical properties

Absorption and fluorescence spectra were acquired using HEPES buffer (50 mM, pH 7.4). Stock solutions (10 mM) of probes **FC‐1** and **FC‐2** were prepared by dissolution in dimethyl sulfoxide (DMSO) prior to dilution in HEPES buffer for analysis. All spectrophotometric and fluorometric measurements were conducted with 5 μM probe solutions in HEPES buffer at 37°C to simulate physiological conditions.

### 4.3 Live cell imaging

SCC7 and 3T3‐L1 cells were seeded into 4‐chamber glass‐bottom dishes at a density of 1.2 × 10^4^ cells per well and cultured under standard conditions for 24 h. Following incubation, the cells were treated with 5 μM **FC‐1** for increasing durations (0, 15, 30, or 60 min). Subsequent to treatment, cells were washed three times with sterile Dulbecco's phosphate‐buffered saline (DPBS) and immediately imaged via CLSM.

To specifically inhibit FAPα and CTSC enzymatic activities, SCC7 cells were pre‐incubated with either 10 μM FAPα‐specific inhibitor SP13786 for 30 min or 20 μM CTSC inhibitor E‐64 for 1 h. Following pretreatment, cells were incubated with 5 μM **FC‐1** for a further 1 h. After three sequential washes with DPBS, the cellular fluorescence was analyzed via CLSM.

### 4.4 In vivo imaging

All animal procedures were conducted in compliance with the guidelines approved by the Animal Ethics Committee of the College of Biology at Hunan University. Female BALB/c and BALB/c nude mice were utilized for all experimental protocols. Prior to imaging, mice were anesthetized via inhalation of 5% isoflurane in 100% oxygen. For tumor imaging, mice were intratumorally administered probe **FC‐1** (50 μL, 100 μM in PBS, containing 20% DMSO, pH 7.4) and subsequently imaged using a Caliper VIS Lumina XR small‐animal in vivo optical imaging system with an excitation wavelength (*λ*_ex_) of 710 nm.

## CONFLICT OF INTEREST STATEMENT

The authors declare no conflicts of interest.

## ETHICS STATEMENT

All animal procedures were performed in accordance with protocol No. SYXK (Xiang) 2023‐0010 approved by the Laboratory Animal Center of Hunan and experiments were approved by the Animal Ethics Committee of College of Biology (Hunan University).

## Supporting information

Supporting Information S1

## Data Availability

All data generated or analyzed during this study are included in this published article.
