# Supplementary material for: Enzyme‐activatable dual‐locked fluorescent probe for precision imaging of cutaneous squamous cell carcinoma
Source: Smart Mol. 2025 Sep 25;3(3):e70018. doi: 10.1002/smo2.70018 (PMC12483136; doi:10.1002/smo2.70018)
Supplement: Supplementary file 1 — Supporting Information S1 [file SMO2-3-e70018-s001.docx]

Supporting Information

Enzyme-Activatable Dual-Locked Fluorescent Probe for Precision Imaging of Cutaneous Squamous Cell Carcinoma

Yanhua Li^1†^, Shan Zuo^1†^, Yushi Chen^2^, Junliang Zhou^1^*, Ling Shi^1^*, Lin Yuan^1^*

^1^State Key Laboratory of Chemo and Biosensing, College of Chemistry and Chemical Engineering, Hunan University, Changsha, Hunan 410082, China

^2^MOE Key Laboratory for Analytical Science of Food Safety and Biology, Fujian Provincial Key Laboratory of Analysis and Detection Technology for Food Safety, College of Chemistry, Fuzhou University, Fuzhou, Fujian 350108, China.

*E-mail: lyuan@hnu.edu.cn (L. Yuan), shiling@hnu.edu.cn (L. Shi), zjl1046@hnu.edu.cn (J. Zhou)

^†^Y. Li and S. Zuo contributed equally to this work.

**Table of Contents**

[**1. General Information** 2](#_Toc193052072)

[**2. Synthesis** 4](#_Toc193052073)

[**3. Supplementary data** 8](#_Toc193052074)

[**4. Copies of NMR Spectra** 10](#_Toc193052075)

**5. Reference** [15](#_Toc193052075)

# **1. General Information**

**Materials and Measurements**

Unless otherwise stated, chemical reagents for synthesis were obtained from commercial suppliers (including Bide Pharmaceutical Technology, Energy Chemical, and Shanghai Titan Technology Company Limited) and were used in the whole experiment without further purification. Cathepsin C (CTSC) was obtained from Sigma. Fibroblast activation protein α (FAPα) was obtained from Sino Biological Inc. Column chromatography silica gel (200-300 mesh) and TLC analysis were purchased from Yantai Jiangyou Silica Gel Development Company Limited. ^1^H NMR and ^13^C NMR spectra were recorded on a Bruker-400 spectrometer with an internal standard (TMS). Mass spectra were performed using an LCQ Advantage ion trap mass spectrometer (Thermo Finnigan) and matrix-assisted layer desorption/ionizationtime-of-flight mass spectrometry (MALDI-TOF/MS, UltrafleXtreme, Bruker). Absorption and fluorescence spectroscopic studies were performed in a UV-1800 ultraviolet and visible spectrophotometer (Shimadzu Corporation, Japan) and an Edinburgh spectrofluorometer FS5 (Edinburgh Innovations, Germany). Cell imaging was performed on Nikon A1 plus confocal microscope (Nikon, Japan). Isoflurane was bought from QingMu Biotechnology (Wuhan, China) Co., Ltd. 5-week-old BALB/c and BALB/c-nude female mice were provided by Hunan SJA Laboratory Animal Company Limited. In vivo imaging was carried out on an IVIS Lumina XR (IS1241N6071) imaging system and a FUSION FX. EDGE. imaging system (Changsha Hemao Instrument Equipment Company Limited).

**Absorption and Fluorescence Analysis**

Absorption and fluorescence spectra were measured in HEPES (50 mM, pH 7.4). The probes for absorption and fluorescence analysis were dissolved in DMSO to get 10 mM stock solution. CTSC and FAPα were dissolved into deionized water to form an aqueous solution and were divided into several parts for daily experiments. FC-1 and FC-2 was added into 200 μL aqueous solution (HEPES/DMSO = 99/1, 50 mM, pH 7.4) containing different concentrations of targets. Then, the mixture solution was incubated at 37 ℃ for 3 h before testing. The concentrations of analytes selected for the selectivity experiments are followed: ARS (9 U/L), Granzyme B (1 ng/μL), LTA4H (1.0 μg/mL), caspase-3 (75 ng/mL), NOQ1 (500 ng/mL), CTSB (75 U/L), ALP (50 U/L).

**High-Performance Liquid Chromatography Assay**

The gradient consisted of five parts: 0-4 min, 95% A-5% B; 4-15 min, from 95% A-5% B to 5% A-95% B; 15-20 min, 5% A-95% B; 20-25 min, from 5% A-95% B to 95% A-5% B; 25-26 min, 95% A-5% B. Phase A: H_2_O containing 0.1% trifluoroacetic acid (TFA); Phase B: Methanol. Other high-performance liquid chromatography (HPLC) conditions were as follows: temperature of 37 °C; flow rate of 1 mL/min; and monitoring wavelength of 660 nm.

**Cell culture**

SCC7 cells and 3T3-L1 cells were cultured in RPMI 1640 medium supplemented with 10% FBS, 1% penicillin and 1% streptomycin.

**Cytotoxicity assay**

The cytotoxicity was measured by methyl thiazolyl tetrazolium (MTT) assay. Cells were seeded into 96-well plate at 1×10^4^/well in log phase. Then cells were treated with various compounds for 24 h. MTT solution (5 mg/mL) was added and incubated for another 4 h. Finally, all the solutions were removed, and 200 μL dimethyl sulfoxide (DMSO) was added to dissolve the formazan. The absorbance at 490 nm was measured by the microplate reader (APC-A700-A detector).

**Animal model**

All animal procedures were performed in accordance with licence No. SYXK (Xiang) 2023-0010 approved by the Laboratory Animal Center of Hunan and experiments were approved by the Animal Ethics Committee of the College of Biology (Hunan University). Mice were kept in a pathogen-free environment and housed in sterile cages with airflow hoods. To generate the SCC7-bearing mice model, 5×10^7^ SCC7 cells in 50 μL serum-free medium were subcutaneously injected in the right flanks of each mouse. The diameter of tumors is ca. 3 mm. Keloid models were established at the tumor excision site, with complete wound healing achieved approximately 5 days post-surgery.

# **2. Synthesis**

**Scheme S1**. Synthesis of **FC-1** and **FC-2**.

**Synthesis of compound** **3**

According to method in the literature,^1^ crude Ac-Gly-Pro-Gly-Phe (1.70 g) was weighed in a 100 mL round-bottomed flask, 25 mL of tetrahydrofuran was added and allowed to dissolve, followed by the addition of 4-aminobenzyl alcohol (549 mg, 4.46 mmol) reacted with 2-ethoxy-1-ethoxycarbonyloxy-1,2-dihydroquinoline (EEDQ (2.00 g, 8.08 mmol) to the solution and reacted overnight at room temperature. At the end of the reaction, the reaction system was filtered to give a white solid **3** (1.20 g). ^1^H NMR (400 MHz, Methanol-*d*_4_) δ 7.50 (d, *J* = 7.8 Hz, 2H), 7.36 – 7.12 (m, 7H), 4.70 (t, *J* = 7.3 Hz, 1H), 4.55 (s, 2H), 4.45 – 4.32 (m, 1H), 4.13 – 3.97 (m, 2H), 3.95-3.90 (m, 1H), 3.74 (d, *J* = 16.8 Hz, 1H), 3.69 – 3.57 (m, 2H), 3.31 (s, 1H), 3.26-3.21 (m, 1H), 3.09-3.03 (m, 1H), 2.29 – 2.15 (m, 1H), 2.15 – 2.06 (m, 1H), 2.07 – 1.94 (m, 2H), 1.89 (s, 3H). ^13^C NMR (101 MHz, Methanol-*d*_4_) δ 175.4, 173.6, 171.5, 170.0, 138.9, 138.5, 138.4, 130.4, 129.5, 128.5, 127.8, 121.5, 64.8, 62.5, 57.2, 47.8, 43.8, 43.0, 39.0, 30.4, 26.0, 22.4. MALDI-TOF MS: (m/z): [M+Na]^+^ Calcd. for [C_27_H_33_N_5_O_6_Na]^+^: 546,232; found 546.038.

**Synthesis of compound FC-1**

Compound **3** (200 mg) was charged into a 50 mL round-bottomed flask, dissolved in anhydrous tetrahydrofuran (4 mL) under sonication, and cooled to 0 °C with stirring. Triphenylphosphine (55 μL, 0.58 mmol) was added dropwise, and the reaction was maintained at 0 °C for 2 h. Upon completion, excess triphenylphosphine was quenched by dropwise addition of saturated aqueous NaHCO₃ solution. The mixture was extracted with ethyl acetate (3 × 20 mL) and water, and the combined organic phases were dried over anhydrous Na₂SO₄. The solvent was removed under reduced pressure to yield a yellowish crude product **5** (306 mg).

HD-OH (86.7 mg, 0.20 mmol) was dissolved in anhydrous acetonitrile (3 mL) in a 50 mL round-bottomed flask. *N,N*-diisopropylethylamine (49.7 μL, 0.38 mmol) and the crude product **5** (140 mg) were added sequentially. The mixture was heated to 50 °C and stirred for 12 h. The reaction was quenched with dichloromethane (20 mL) and water (10 mL). The organic layer was separated, washed with brine, and dried over anhydrous Na₂SO₄. After concentration under reduced pressure, the residue was purified by silica gel column chromatography using a gradient of dichloromethane/methanol (100:4, v/v) as the eluent, affording the desired compound as a blue solid (25.8 mg, 13.7% yield). ^1^H NMR (400 MHz, Methanol-*d*_4_) δ 8.68 (d, *J* = 14.5 Hz, 1H), 7.73 (d, *J* = 6.7 Hz, 1H), 7.66 (s, 1H), 7.64 (s, 1H), 7.59 (s, 2H), 7.51 (d, *J* = 9.8 Hz, 2H), 7.47 (s, 2H), 7.23 (s, 5H), 7.15 (s, 2H), 6.53 (d, *J* = 15.3 Hz, 1H), 5.31 (s, 2H), 4.68 (s, 1H), 4.42 (d, *J* = 6.3 Hz, 2H), 4.35 (s, 1H), 4.18 – 3.93 (m, 2H), 3.91 – 3.72 (m, 2H), 3.72 – 3.53 (m, 2H), 3.24 (s, 2H), 3.10 – 2.99 (m, 1H), 2.73-2.69 (m, 4H), 2.11 – 2.00 (m, 2H), 1.96 (d, *J* = 12.2 Hz, 2H), 1.91 (s, 2H), 1.87 (s, 3H), 1.83 (s, 6H), 1.48 (t, *J* = 5.9 Hz, 3H). ^13^C NMR (101 MHz, Methanol-*d*_4_) δ 177.8, 174.0, 172.2, 170.2, 168.8, 160.5, 156.6, 152.6, 145.9, 142.5, 141.0, 138.1, 137.1, 131.8, 131.4, 129.0, 128.4, 128.1, 127.7, 127.6, 127.4, 126.4, 122.6, 120.2, 120.1, 116.0, 114.5, 112.6, 104.1, 101.5, 70.9, 61.2, 56.0, 50.9, 42.5, 41.6, 40.3, 37.5, 31.7, 29.4, 29.0, 26.9, 24.6, 23.6, 22.4, 21.0, 13.1, 11.7. MALDI-TOF MS: (m/z): [M]^+^ Calcd. for [C_54_H_58_ClN_6_O_7_]^+^: 937.405; found 937.327.

**Synthesis of compound** **4**

According to method in the literature,^1^ crude Cbz-Gly-Pro-Gly-Phe (2 g) was weighed in a 100 mL round-bottomed flask, 25 mL of tetrahydrofuran was added and allowed to dissolve, followed by the addition of 4-aminobenzyl alcohol (562 mg, 4.56 mmol) reacted with 2-ethoxy-1-ethoxycarbonyloxy-1,2-dihydroquinoline (EEDQ (2.05 g, 8.28 mmol) to the solution and reacted overnight at room temperature. At the end of the reaction, the reaction system was filtered to give a white solid **4** (580 mg). ^1^H NMR (400 MHz, Methanol-*d*_4_) δ 7.50 (d, *J* = 8.1 Hz, 2H), 7.28-7.16 (m, 12H), 4.99 – 4.88 (m, 2H), 4.68 (t, *J* = 7.4 Hz, 1H), 4.54 (s, 2H), 4.47 – 4.31 (m, 1H), 4.09 – 3.84 (m, 3H), 3.83 – 3.54 (m, 3H), 3.24-3.18 (m, 1H), 3.09-3.03 (m, 1H), 2.28 – 2.16 (m, 1H), 2.15 – 2.07 (m, 1H), 2.03-1.98 (m, 2H). ^13^C NMR (101 MHz, Methanol-*d*_4_) δ 175.4, 171.5, 171.5, 170.6, 158.9, 138.8, 138.5, 138.4, 138.0, 130.5, 129.4, 129.4, 129.0, 128.8, 128.5, 127.7, 121.5, 67.8, 64.8, 62.6, 57.3, 47.8, 44.2, 43.8, 39.0, 30.3, 26.0. MALDI-TOF MS: (m/z): [M+Na]^+^ Calcd. for [C_33_H_37_N_5_O_7_Na]^+^: 638.258; found 638.070.

**Synthesis of compound FC-2**

Compound **4** (160 mg, 0.31 mmol) was charged into a 50 mL round-bottomed flask, dissolved in anhydrous tetrahydrofuran (4 mL) under sonication, and cooled to 0 °C with stirring. Triphenylphosphine (38 μL, 0.40 mmol) was added dropwise, and the reaction was maintained at 0 °C for 2 h. Upon completion, excess triphenylphosphine was quenched by dropwise addition of saturated aqueous NaHCO₃ solution. The mixture was extracted with ethyl acetate (3 × 20 mL) and water, and the combined organic phases were dried over anhydrous Na₂SO₄. The solvent was removed under reduced pressure to yield a yellowish crude product **6** (290 mg).

HD-OH (76.9 mg, 0.18 mmol) was dissolved in anhydrous acetonitrile (3 mL) in a 50 mL round-bottomed flask. *N,N*-diisopropylethylamine (29 μL, 0.25 mmol) and the crude product **6** (80 mg) were added sequentially. The mixture was heated to 50 °C and stirred for 12 h. The reaction was quenched with dichloromethane (20 mL) and water (10 mL). The organic layer was separated, washed with brine, and dried over anhydrous Na₂SO₄. After concentration under reduced pressure, the residue was purified by silica gel column chromatography using a gradient of dichloromethane/methanol (100:4, v/v) as the eluent, affording the desired compound as a blue solid **FC-2** (29.3 mg, yield 15.8%). ^1^H NMR (400 MHz, Methanol-*d*_4_) δ 8.63 (d, *J* = 14.9 Hz, 1H), 7.73 (d, *J* = 7.3 Hz, 1H), 7.65 (d, *J* = 8.0 Hz, 2H), 7.58 (t, *J* = 9.7 Hz, 2H), 7.50 (d, *J* = 7.2 Hz, 2H), 7.45 (d, *J* = 8.2 Hz, 2H), 7.21 (s, 2H), 7.17 (s, 3H), 7.12 (d, *J* = 8.8 Hz, 7H), 6.51 (d, *J* = 14.9 Hz, 1H), 5.33 (s, 2H), 4.83 (d, *J* = 9.7 Hz, 3H), 4.65 (t, *J* = 7.5 Hz, 1H), 4.41 (d, *J* = 7.1 Hz, 2H), 4.31 (s, 1H), 3.95 (d, *J* = 11.2 Hz, 2H), 3.88-3.80 (m, 1H), 3.74-3.64 (m, 1H), 3.65 – 3.59 (m, 1H), 3.57 (s, 1H), 3.17 (dd, *J* = 13.8, 6.5 Hz, 1H), 3.00 (dd, *J* = 13.3, 8.5 Hz, 1H), 2.76 – 2.57 (m, 4H), 2.19 (s, 1H), 2.07 (s, 1H), 1.95 (d, *J* = 2.9 Hz, 2H), 1.89 (d, *J* = 5.8 Hz, 2H), 1.82 (d, *J* = 9.5 Hz, 6H), 1.47 (d, *J* = 7.1 Hz, 3H).^13^C NMR (101 MHz, Methanol-*d*_4_) δ 187.9, 174.0, 170.1, 169.2, 164.6, 160.6, 157.4, 156.7, 156.6, 152.5, 145.9, 142.5, 141.0, 138.2, 136.4, 131.8, 131.5, 129.5, 129.0, 128.4, 128.0, 128.0, 127.7, 127.5, 127.4, 126.3, 122.7, 120.2, 116.0, 114.4, 112.6, 101.6, 66.3, 61.3, 56.1, 53.4, 50.9, 48.2, 42.8, 42.5, 40.3, 37.6, 31.7, 30.3, 29.4, 29.2, 29.1, 28.9, 28.9, 28.7, 27.0, 26.9, 26.7, 24.7, 22.3, 20.1, 13.0, 11.6. MALDI-TOF MS: (m/z): [M]^+^ Calcd. for [C_60_H_62_ClN_6_O_8_]^+^: 1029.431; found 1029.348.

**Scheme S2**. Synthesis of **F-1**.

**Synthesis of compound F-1**

The synthesis of compound **7** was obtained from the literature.^2^

Bis(trichloromethyl)carbonate (BTC, 149.8 mg, 0.50 mmol) was added to a 25 mL round-bottom flask under a nitrogen atmosphere at 0 °C. Anhydrous dichloromethane (DCM) was introduced via syringe. HD-OH (80.15 mg, 0.18 mmol) was dissolved in anhydrous DCM and transferred to the reaction mixture via syringe. After 30 min of stirring, DIPEA (108 mg, 0.84 mmol; followed by 43.5 mg, 0.33 mmol) was added dropwise via syringe, and stirring continued for an additional 30 min. Compound **7** (61 mg, 0.16 mmol), dissolved in anhydrous DCM, was added via syringe, and the reaction proceeded under nitrogen for 5 h. The reaction mixture was concentrated under reduced pressure, and the residue was partitioned between DCM and water. The organic layer was dried over anhydrous Na_2_SO_4_, filtered, and concentrated in vacuo to afford a crude solid. The crude product was purified by gradient silica gel chromatography (DCM/MeOH, 100:6 v/v) to yield **F-1** as a blue solid (17.0 mg, 9.6%). ^1^H NMR (400 MHz, Methanol-d4) δ 8.76 (d, J = 15.2 Hz, 1H), 8.59 (t, J = 15.7 Hz, 1H), 7.60 (d, J = 5.1 Hz, 3H), 7.56 (d, J = 3.9 Hz, 3H), 7.32 – 7.30 (m, 3H), 7.25 (s, 4H), 7.18 (d, J = 6.1 Hz, 2H), 6.63 (d, J = 15.2 Hz, 1H), 5.08 – 5.04 (m, 4H), 4.52 (dd, J = 8.5, 3.2 Hz, 2H), 4.45 (d, J = 7.3 Hz, 3H), 3.99 (d, J = 11.0 Hz, 3H), 3.61 (d, J = 9.9 Hz, 6H), 3.07 – 3.04 (m, 2H), 3.02 (s, 1H), 2.99 (d, J = 5.6 Hz, 2H), 2.96 (s, 1H), 2.74 (d, J = 4.7 Hz, 2H), 2.70 – 2.67 (m, 2H), 2.01 (d, J = 5.5 Hz, 4H), 1.92 – 1.89 (m, 2H), 1.80 (d, J = 2.8 Hz, 4H), 1.74 – 1.72 (m, 2H). ^13^C NMR (101 MHz, Methanol-d4) δ 178.9, 172.2, 169.8, 160.4, 160.1, 158.6, 157.7, 154.4, 152.2, 147.5, 147.3, 141.8, 137.8, 130.4, 130.0, 129.6, 129.0, 128.6, 128.4, 125.8, 123.5, 120.5, 120.4, 120.2, 114.0, 112.7, 67.3, 62.0, 54.4, 47.4, 43.7, 41.6, 36.1, 32.7, 30.3, 30.2, 30.1, 29.9, 27.7, 27.6, 23.3, 14.1, 12.8. MALDI-TOF MS: (m/z): [M]^+^ Calcd. for [C_55_H_60_ClN_6_O_9_]^+^: 983.410; found 983.325.

# **3. Supplementary data**


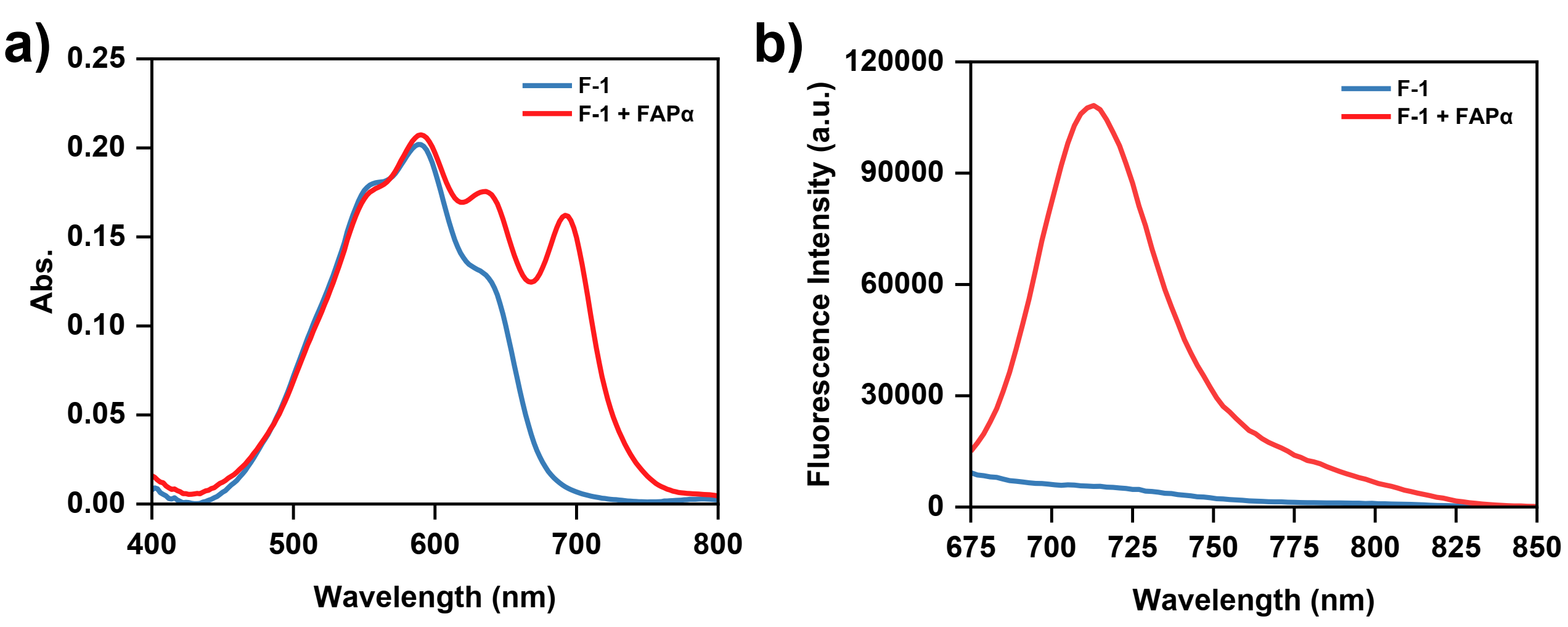


**Figure S1.** UV/Vis absorption (a) and fluorescence (b) spectra of 5 μM **F-1** were recorded in the absence and presence of FAPα (1.2 mU/mL) after incubation for 30 min at 37 °C in HEPES buffer (50 mM, pH 7.4).


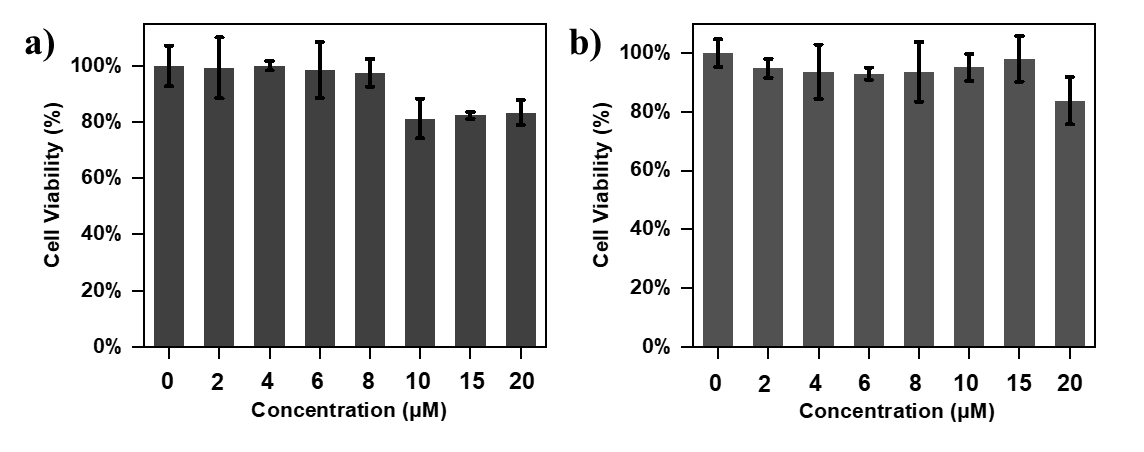


**Figure S2.** Cytotoxicity assay of **FC-1** (a) and **FC-2** (b) in SCC7 cells


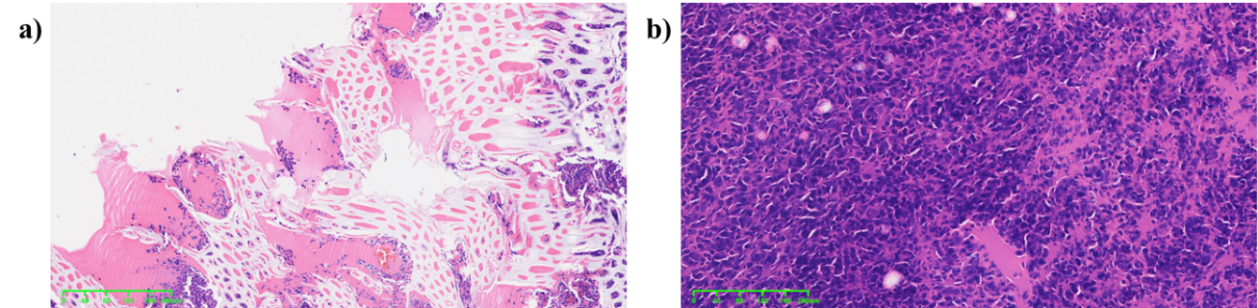


**Figure S3**. H&E staining of keloid tissue (a) and tumor (b) tissue. scale bar: 200 μm.

# **4. Copies of NMR Spectra**


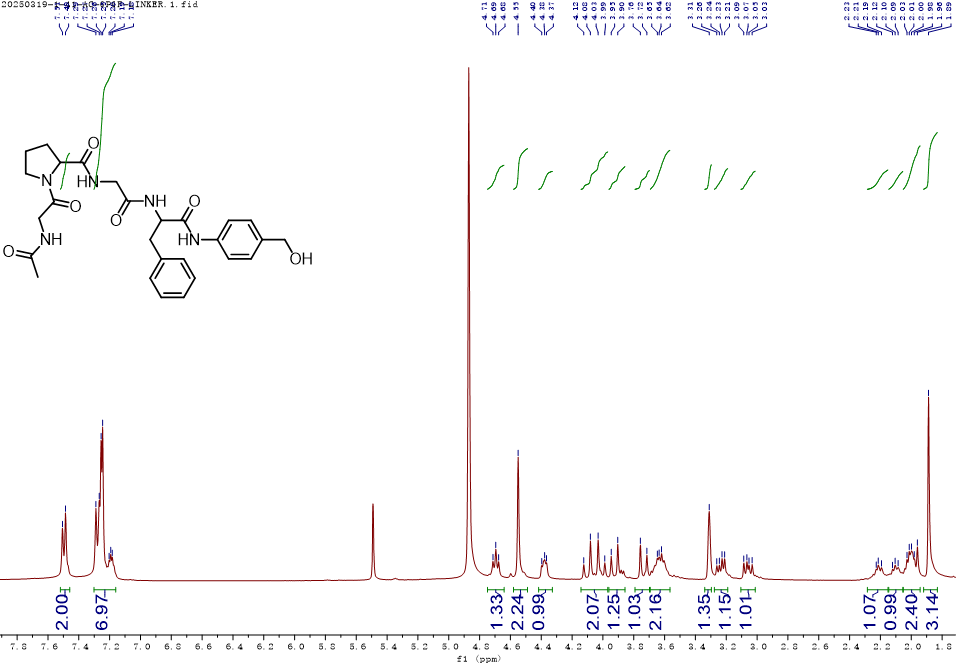


**Figure S4**. ^1^H NMR (400 MHz) spectrum of compound **3** in Methanol-*d*_4_.


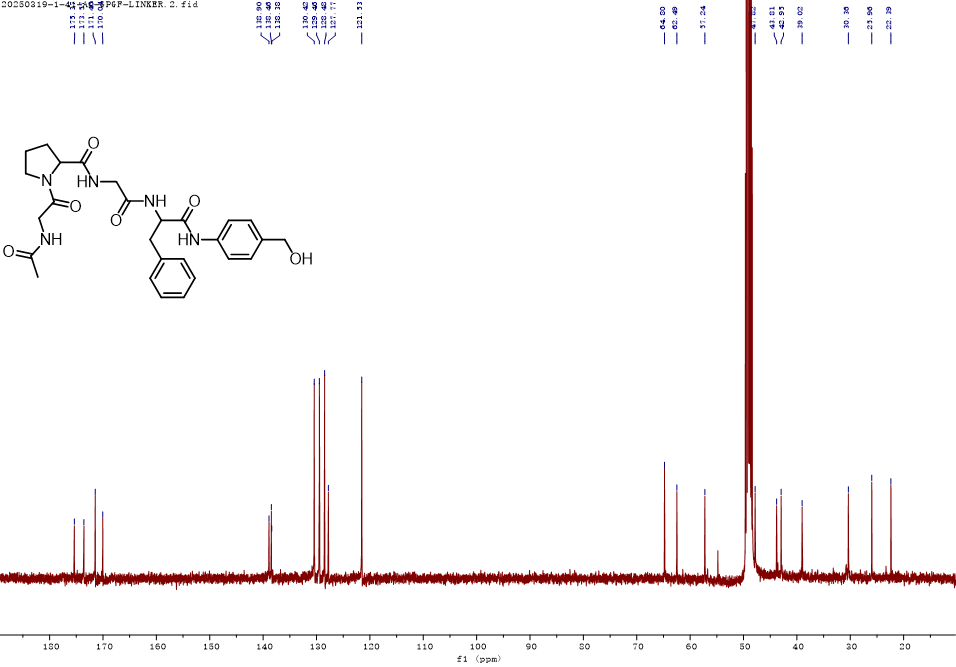


**Figure S5**. ^13^C NMR (101 MHz) spectrum of compound **3** in Methanol-*d*_4_.


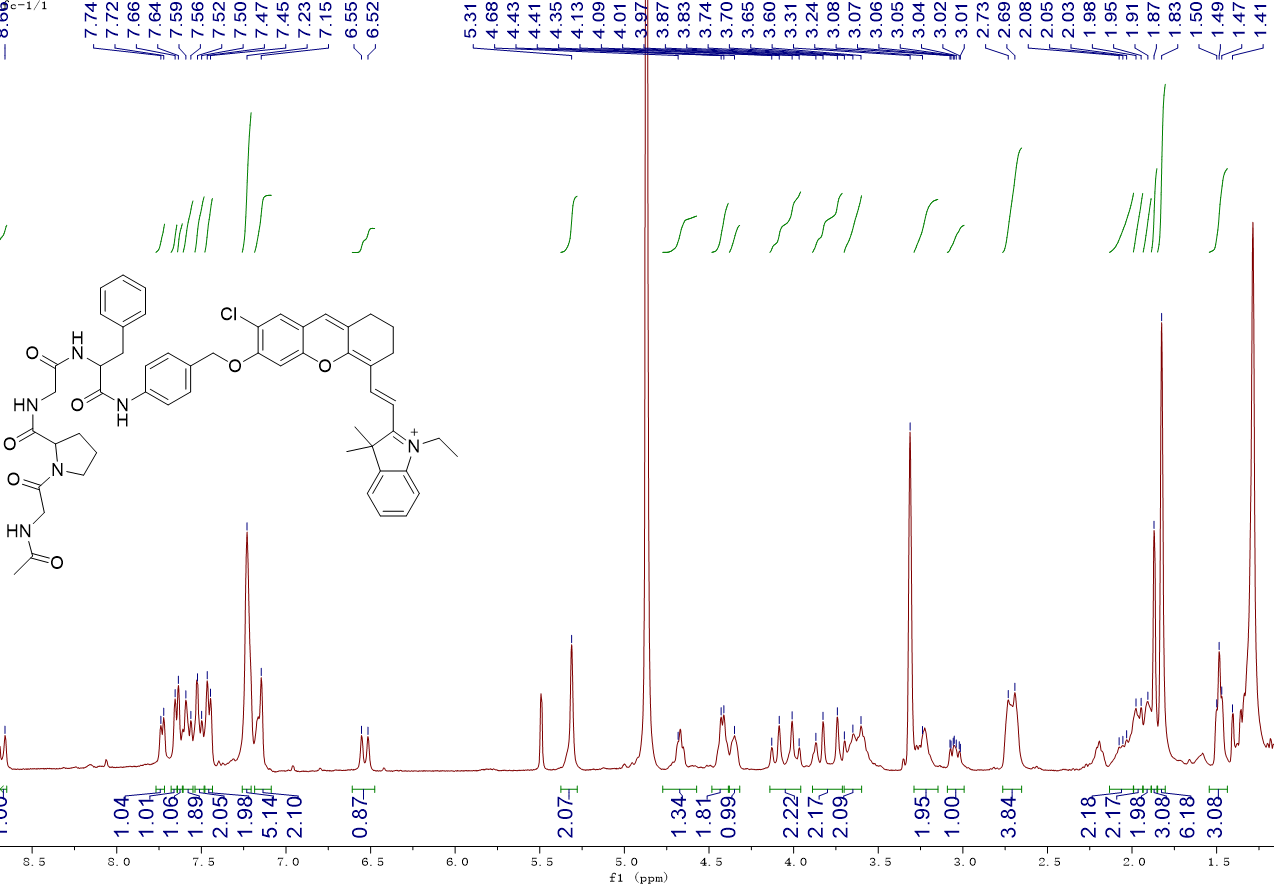


**Figure S6**. ^1^H NMR (400 MHz) spectrum of compound **FC-1** in Methanol-*d*_4_.


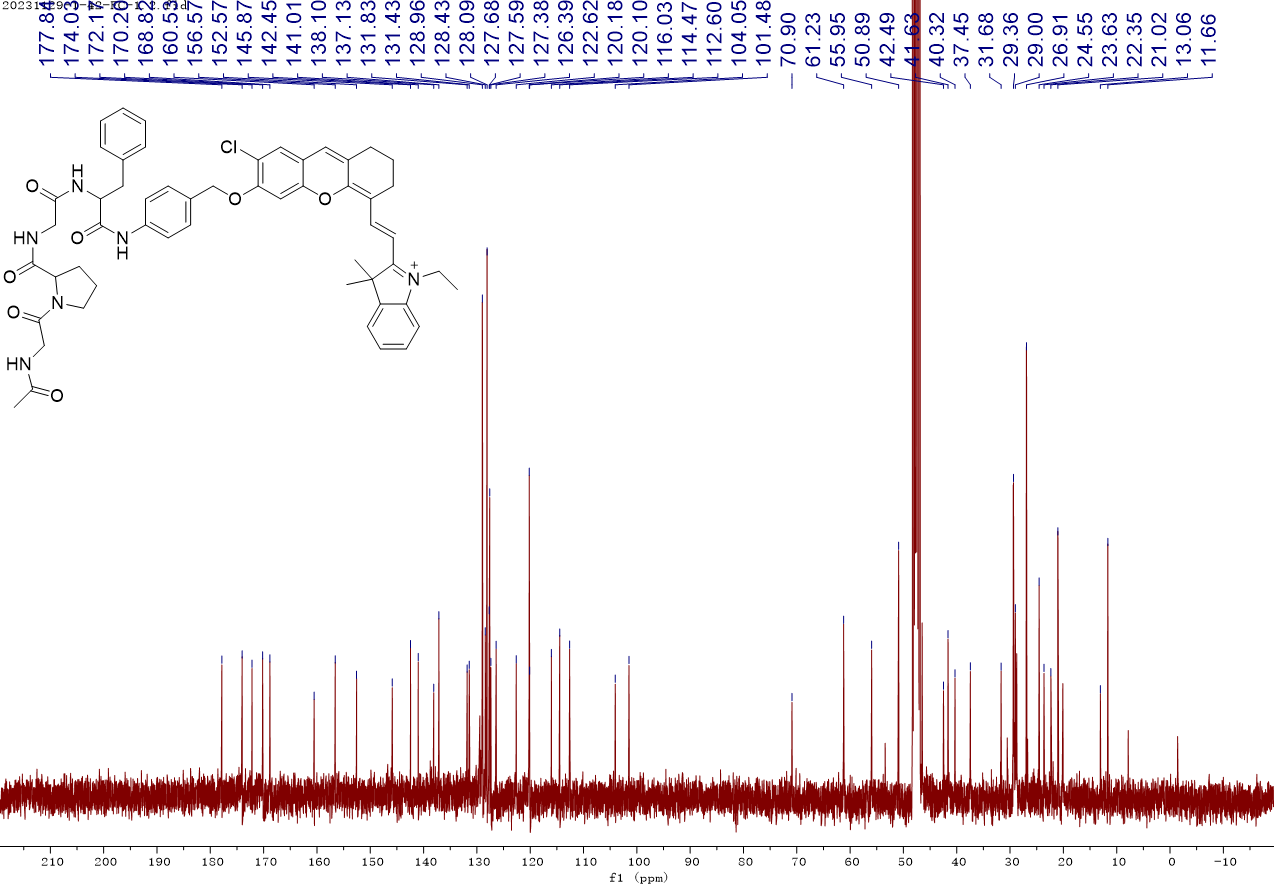


**Figure S7**. ^13^C NMR (101 MHz) spectrum of compound **FC-1** in Methanol-*d*_4_.


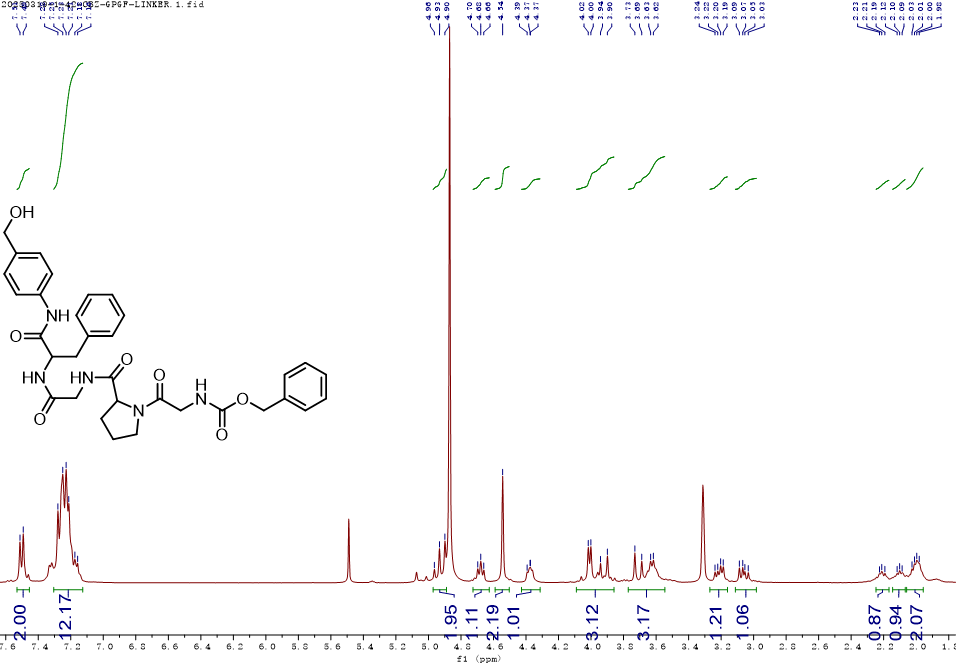


**Figure S8**. ^1^H NMR (400 MHz) spectrum of compound **4** in Methanol-*d*_4_.


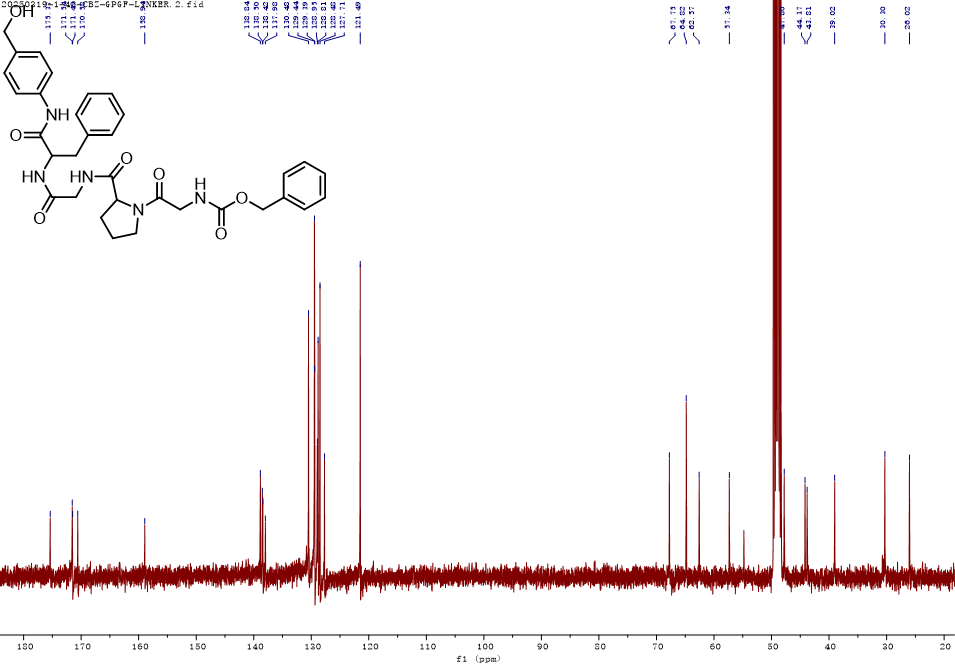


**Figure S9**. ^13^C NMR (101 MHz) spectrum of compound **4** in Methanol-*d*_4_.


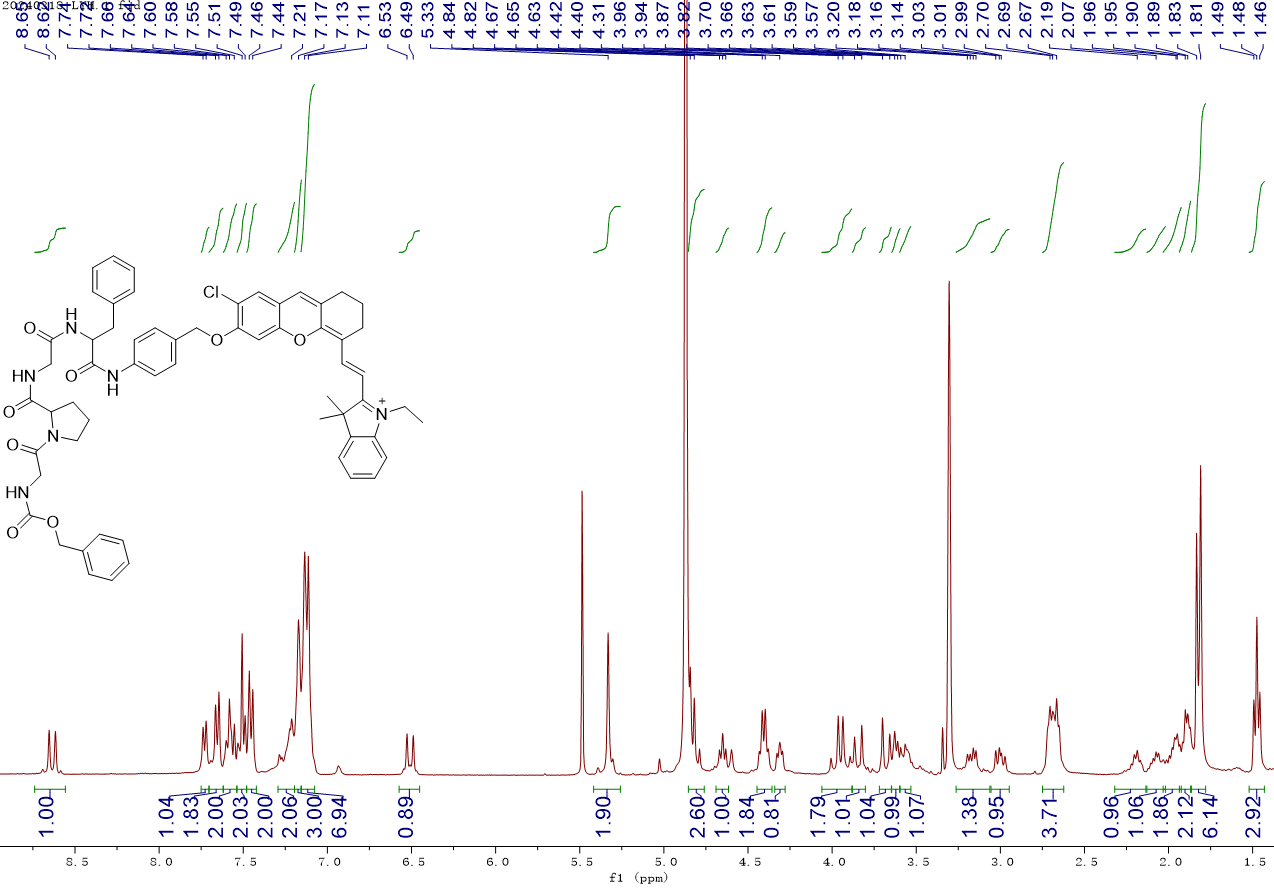


**Figure S10**. ^1^H NMR (400 MHz) spectrum of compound **FC-2** in Methanol-*d*_4_.


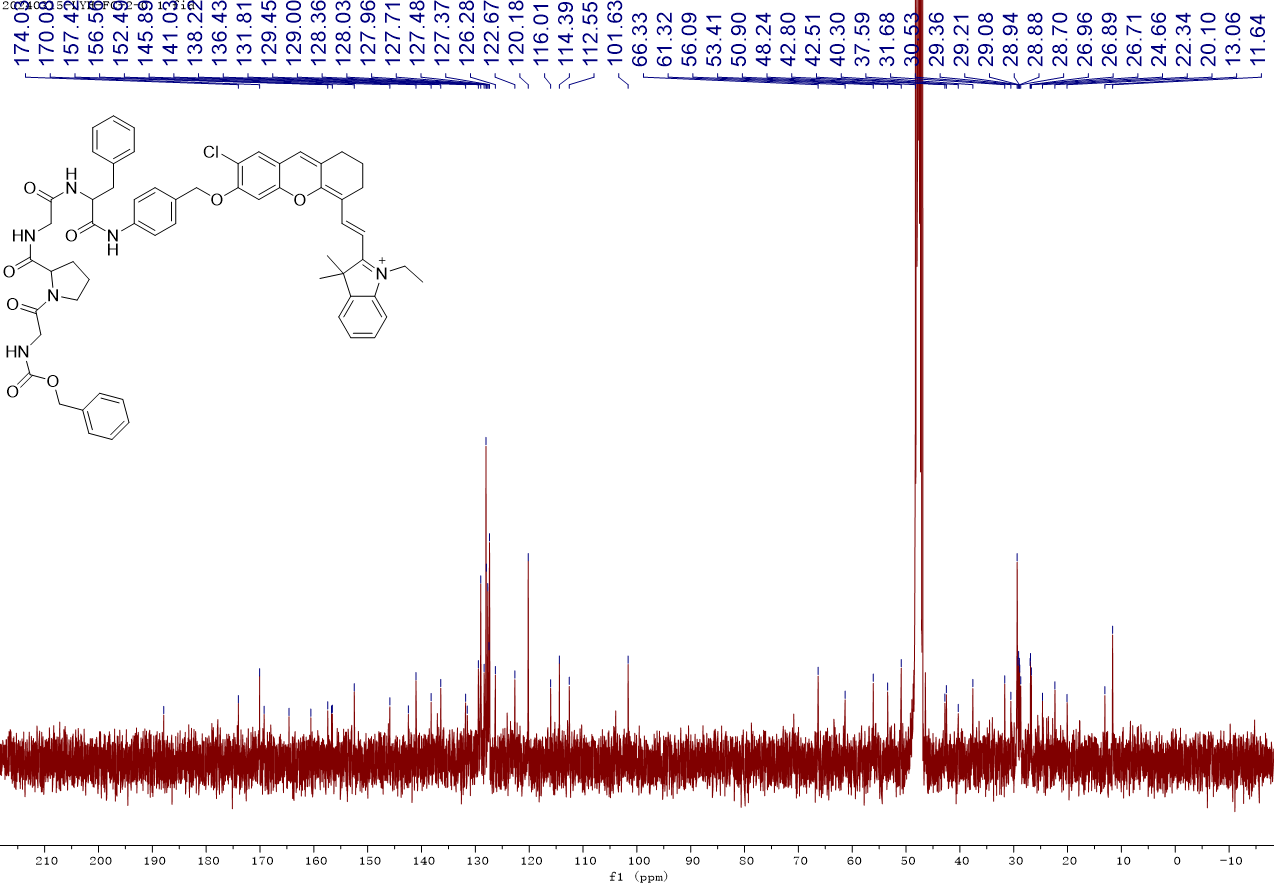


**Figure S11**. ^13^C NMR (101 MHz) spectrum of compound **FC-2** in Methanol-*d*_4_.


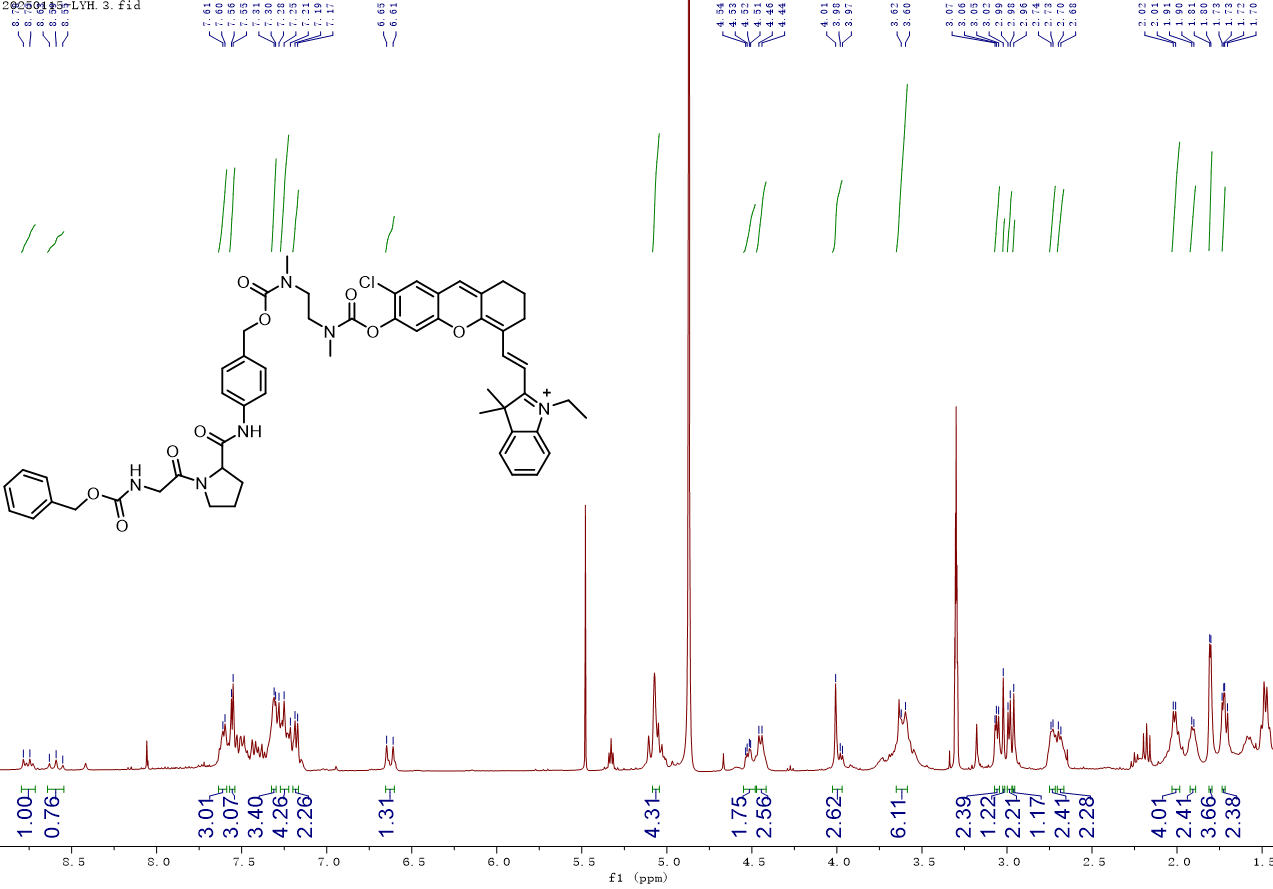


**Figure S12**. ^1^H NMR (400 MHz) spectrum of compound **F-1** in Methanol-*d*_4_


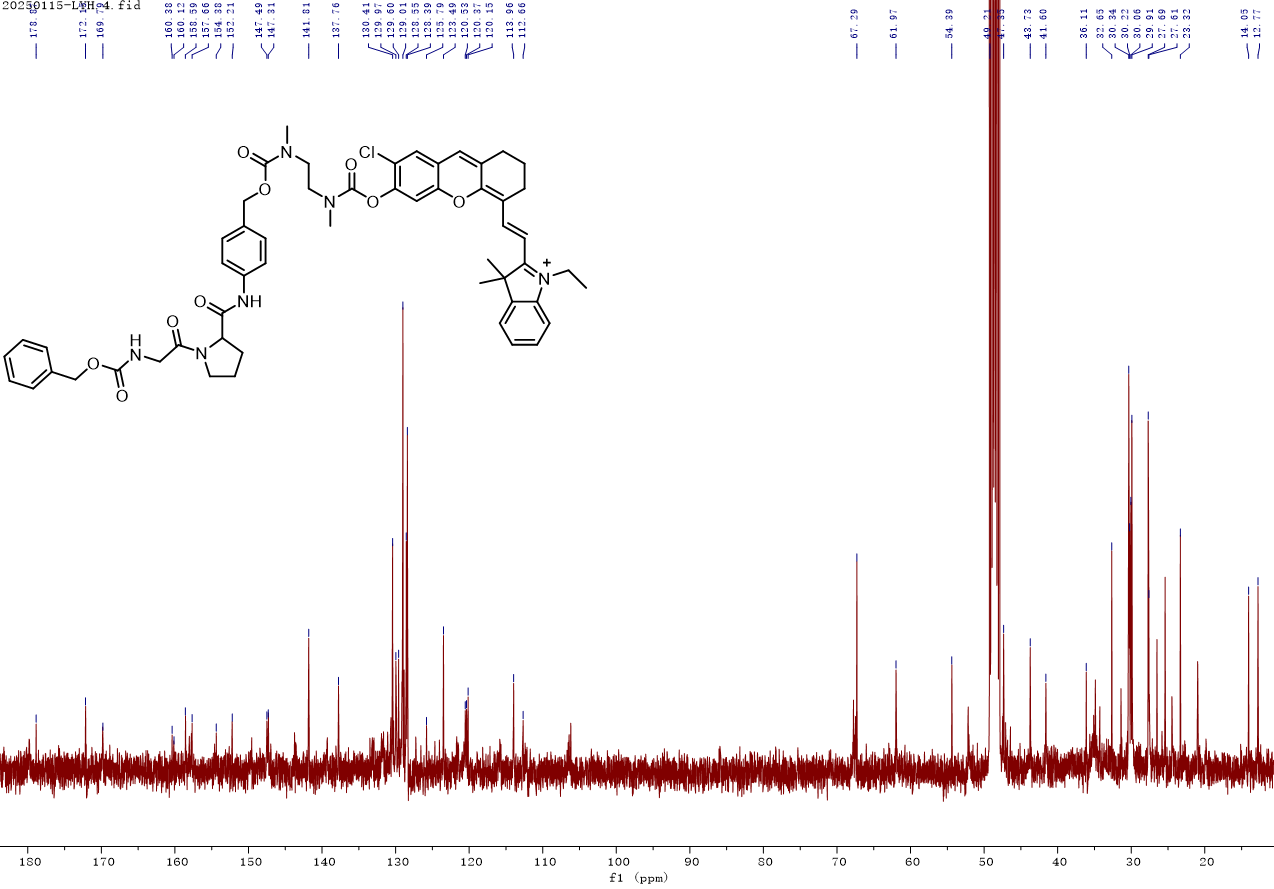


**Figure S13**. ^13^C NMR (101 MHz) spectrum of compound **F-1** in Methanol-*d*_4_.

# **5. Reference**

1. Wang, X.; He, S.; Cheng, P.; Pu, K., A Dual-Locked Tandem Fluorescent Probe for Imaging of Pyroptosis in Cancer Chemo-Immunotherapy. *Adv. Mater.* **2023,** *35* (10), 2206510.

2. Miao, Q.; Yeo, D. C.; Wiraja, C.; Zhang, J.; Ning, X.; Xu, C.; Pu, K., Near-Infrared Fluorescent Molecular Probe for Sensitive Imaging of Keloid. *Angew. Chem. Int. Ed.* **2018,** *57* (5), 1256-1260.
